# Association of Maternal Diet during Pregnancy and Metabolite Profile in Cord Blood

**DOI:** 10.3390/biom12101333

**Published:** 2022-09-21

**Authors:** Carla P. Harris, Carlana Ramlochansingh, Olaf Uhl, Hans Demmelmair, Joachim Heinrich, Berthold Koletzko, Marie Standl, Elisabeth Thiering

**Affiliations:** 1 Institute of Epidemiology, Helmholtz Zentrum München GmbH, German Research Center for Environmental Health, 85764 Neuherberg, Germany; 2 Dr. von Hauner Children’s Hospital, University Hospital, LMU Munich, 80337 Munich, Germany; 3 Institute of Medical Informatics, Biometry and Epidemiology (IBE), LMU Munich, 81377 Munich, Germany; 4 Institute and Outpatient Clinic for Occupational, Social and Environmental Medicine, LMU University Hospitals, 80336 Munich, Germany; 5 Allergy and Lung Health Unit, Melbourne School of Population and Global Health, The University of Melbourne, Parkville, VIC 3010, Australia

**Keywords:** targeted metabolomics, maternal diet, dietary patterns, pregnancy, cord blood, early programming, epidemiology

## Abstract

Cord blood metabolites can be predictive of long-term disease risk, but how levels of different metabolites might vary with respect to maternal diet is not well understood. The aim of this study was to evaluate the associations of different dietary patterns during pregnancy with cord blood metabolites (including glycerophospholipid fatty acids, polar lipids, non-esterified fatty acids, amino acids, and the sum of hexoses). Participants from the German LISA birth cohort study, with available data on targeted cord blood metabolomics and maternal diet, were included (n = 739). Maternal diet during the last 4 weeks of pregnancy was assessed by a non-quantitative food-frequency questionnaire. Using factor analysis, ten dietary patterns were identified, which were used in linear regression models exploring associations with cord blood metabolites. After correction for multiple hypothesis testing and adjustment for basic covariates, “fish and shellfish” was associated with higher glycerophospholipid fatty acid C20:5 n3 and lower C22:5 n6, whereas the “meat and potato” pattern was directly associated with propionylcarnitine (C3:0). The observed associations highlight potential metabolic pathways involved in the early programming of health and disease through maternal diet, as well as the potential for establishing quantitative biomarkers for dietary patterns of pregnant women.

## 1. Introduction

Maternal diet during pregnancy contributes to fetal growth and development, playing a crucial role in the short- and long-term health of the offspring. It is well established that certain key nutrient deficiencies in the mother can lead to health problems in the offspring, manifesting as impaired physical and/or mental capacities [1,2,3]. Furthermore, it is now widely accepted that many adult-onset chronic diseases may have their origins before birth, with maternal diet hypothesized as a primary programming stimulus [4,5]. For example, reduced birth size resulting from insufficient fetal nutrient supply is associated with a higher risk of chronic diseases in adulthood [6,7,8]. Low birth weight is often followed by increased postnatal weight gain, which in turn has been linked to asthma [9] and reduced lung function in adolescence [10]. In contrast, excessive maternal energy intake increases the risk for high birth weight, leading to increased risk of overweight in later childhood [11,12,13].

Growing evidence also highlights the importance of diet composition to ensure optimal health of the offspring [14]. The human diet typically involves combinations of different foods and nutrients, and thus, their roles must be interpreted in the context of possible correlations and interactions among them. For this, the study of dietary patterns can be highly informative [15]. A number of studies on maternal dietary patterns have observed significant associations with birth outcomes, most often reporting protective effects of vegetables, fruit, whole grains, and fish [16,17,18], and negative effects of diets high in fat, sugars, and processed foods [19,20,21]. Nevertheless, the metabolic pathways through which different dietary patterns may influence fetal development and future chronic diseases are poorly understood. We assume that the maternal diet affects metabolism, and consequently the intrauterine milieu, hence determining metabolic adaptations in the neonate. The cord blood metabolome provides information on the intrauterine environment, which involves the complex interaction of maternal metabolism, placental nutrient transfer, and fetal metabolism [22]. Studies have shown significant associations between maternal metabolic status, such as BMI or dysglycemia, and cord blood metabolites [23,24,25,26]. Others have identified metabolic profiles associated with unfavorable birth outcomes [27,28,29,30]. However, there is limited evidence on the impact of maternal diet on cord blood metabolites [31]. Thus, in the present study, we applied targeted metabolomics in order to investigate the association of maternal dietary patterns during pregnancy with cord blood metabolites. We demonstrated associations with specific metabolites with known roles in lipid signaling, beta-oxidation, and branched-chain amino acid degradation. Ultimately, if confirmed in future studies, these findings could contribute to a better understanding of early disease etiology.

## 2. Materials and Methods

### 2.1. Study Population

Data obtained from participants of the LISA (Influence of Life-Style Factors on the Development of the Immune System and Allergies in East and West Germany) study were used for the present analyses [32]. In summary, 3097 healthy full-term newborns (of which 3 later withdrew consent) were recruited from November 1997 to January 1999 from four regions of Germany (Munich, Leipzig, Wesel, and Bad Honnef). At birth, 4 mL of venous cord blood was collected and centrifuged at 1400× *g* for 10 min. The resulting serum was deep-frozen at −80 °C until metabolomics analysis. Information on maternal diet during the last four weeks of pregnancy was obtained by means of a non-quantitative food-frequency questionnaire, administered to the mothers after birth. Dietary assessment and metabolomics analysis are described in more detail below. Additional information was collected after birth through questionnaires, including gestational age at birth (weeks), smoking during the 3rd trimester of pregnancy (yes; no), maternal education (low: <10 years of education; medium: 10 years; high: >10 years), maternal age (years), maternal pre-pregnancy BMI (kg/m^2^, categorized as underweight: <18.5; normal weight: ≥18.5 and <25; and overweight: ≥25, according to WHO classification [29]), gestational weight gain (kg/month), and birth weight of the neonate (kg). Approval by the local ethics committees (Bavarian Board of Physicians, Board of Physicians of North-Rhine-Westphalia) and written consent from the parents were obtained.

### 2.2. Metabolomics Analysis

Glycerophospholipid fatty acids (GPL-FA), acylcarnitines (AC), diacyl-phosphatidylcholines (PCa), acyl-alkyl-phosphatidylcholines (PCe), sphingomyelins (SM), acyl-lysophosphatidylcholines (LPCa), alkyl-lysophosphatidylcholines (LPCe), non-esterified fatty acids (NEFA), amino acids, and the sum of hexoses (H1), were measured in 750 samples of cord blood serum. Measurements were performed at the laboratory of the Division of Metabolic and Nutritional Medicine, Dr. von Hauner Children’s Hospital, Ludwig-Maximilians-Universität München.

Analysis of GPL-FA was performed by transesterification of GPL-FA into methyl esters and their gas chromatographic separation, flame ionization detection (Agilent 7890A, Agilent Technologies, Waldbronn, Germany), and quantification as previously described [33]. AC, PC, SM, LPC, and the sum of hexoses were analyzed by flow-injection analysis tandem mass spectrometry as previously reported [31]. Briefly, 10 μL of samples were diluted with methanol for protein precipitation, containing internal standards for different lipid groups (D_3_-carnitine C2, D_3_-carnitine C8, D_3_-carnitine C16, ^13^C_6_-D-glucose (all Cambridge Isotope Laboratories, Tewksbury, MA, USA), acyl-lysophosphatidylcholine C13:0, and diacyl-phosphatidylcholine C28:0 (both Avanti Polar Lipids, Alabaster, AL, USA)) and ammonium acetate. After centrifugation, supernatants were injected into a high-performance liquid chromatography system (1200, Agilent, Waldbronn, Germany) coupled to a triple quadrupole mass spectrometer (QTRAP4000, Sciex, Darmstadt, Germany) with an electrospray ionization source. The system was run in multiple reaction monitoring (MRM) mode. NEFA were analyzed as previously described [34]. Briefly, 10 μL of the samples was mixed with 200 µL isopropanol containing internal standard (uniformly labeled palmitic acid U-^13^C16, 98%, Euriso-Top) for protein precipitation. After centrifugation, an aliquot of the supernatant was injected into a high-performance liquid chromatography system (1200, Agilent, Waldbronn, Germany) coupled to a triple quadrupole mass spectrometer (QTRAP4000, Sciex, Darmstadt, Germany) operating in multiple reaction monitoring mode after negative electrospray ionization. Unlike for GPL-FA, the analytical technique applied for AC, PC, SM, LPC, and NEFA is not capable of determining the position of the double bonds and the distribution of carbon atoms between fatty acid side chains. Therefore, these were separated according to chain length and number of double bonds, but not according to the position of double bonds. In the applied nomenclature for these metabolites, CX:Y, X is the length of the carbon chain, and Y is the number of double bonds. ‘a’ indicates that the acyl chain is bound via an ester bond to the backbone, while ‘e’ indicates binding by an ether bond.

Finally, amino acid (AA) analysis was performed as previously reported [35]. Briefly, 10 μL of samples was diluted with the corresponding internal standard reagent. A labeled AA standards set (set A) was mixed with ^15^N_2_-L-asparagine, and indole-D_5_-L-tryptophan (all Cambridge Isotope Laboratories, Tewksbury, MA, USA) and added to the precipitation reagent for internal standardization. After centrifugation, AA were derivatized to AA butyl esters and determined by ion-pair high-performance liquid chromatography (1100, Agilent, Waldbronn, Germany) coupled to a triple quadrupole mass spectrometer (API 2000, Applied Biosystems, Darmstadt, Germany) with an atmospheric pressure chemical ionization source operating in positive ionization mode and multiple reaction monitoring.

Six plasma quality control samples were measured twice, along with the samples per batch. The coefficient of variation (CV) was calculated for each single batch (intra-batch) and for all batches (inter-batch). Regarding intra-batch precision, batches with a CV larger than 30% were excluded for single metabolite measurements, and complete metabolite measurements with an inter-batch CV > 30% were excluded. We report all metabolite concentrations in μmol/L cord blood serum.

### 2.3. Dietary Assessment

Maternal dietary intake during the last 4 weeks of pregnancy was assessed by means of a non-quantitative food-frequency questionnaire (FFQ), administered shortly after childbirth [36]. For the 45 food items included in the FFQ, mothers were asked to report their average consumption frequency over the last 4 weeks. For all food items except milk and yoghurt, this was done by selecting from a choice of five frequency categories, namely: “never or less than 2 times per month”, “2–3 times per month”, “1–2 times per week”, “3–4 times per week”, and “more than 4 times per week”. Milk and yoghurt intakes were quantified according to four categories, defined for milk as “never”, “sometimes”, “up to 0.5 L per day”, and “more than 0.5 L per day”, and for yoghurt as “never”, “sometimes”, “up to 200 g per day”, and “more than 200 g per day”. Subjects were excluded (78 of 3094) if they had missing responses to more than 9 food items, which amounted to 20% of the FFQ. In accordance with recommendations by Willett for data cleaning in nutritional epidemiology [37], sporadic blanks in an otherwise carefully completed FFQ were considered as no consumption of that particular food item, defined as “never or less than 2 times per month” and as “0 L/day” or “0 g/day” for milk and yoghurt, respectively.

### 2.4. Statistical Analysis

All statistical analyses were performed using R version 3.4.2 (R Foundation for Statistical Computing, Vienna, Austria. URL https://www.R-project.org/, first accessed on 1 October 2017) [38]. As a first step, we conducted an exploratory factor analysis to identify maternal dietary patterns, which would then be used in association analysis with cord blood metabolites. Factor analysis seeks to compress several variables into a few underlying factors, based on the degree to which they are correlated with one another. It is hence a useful tool for investigating complex exposures such as habitual diet, going beyond single foods and nutrients, which facilitates interpretation and accounts for possible correlations between foods that are commonly consumed together [39]. Additionally, the method reduces the number of tests to be carried out, limiting the occurrence of chance findings. Firstly, the 45 reported food items were presented in a correlation matrix and the eigenvalues of the matrix calculated for the extraction of factors. The optimal number of factors was decided based on the Kaiser–Guttman rule that states that factors with an eigenvalue greater than 1 should be used [40,41]. The number of factors to retain following this rule was defined by a Non-Graphical Cattell’s Scree test, using the *nScree* function in the package “nFactors” [42]. We then performed a posteriori determination of dietary patterns by maximum likelihood factor analysis with varimax rotation, using the *factanal* function in the package “stats” [38]. Varimax rotation is an orthogonal rotation of the factor axes that maximizes variance of the squared loadings of a factor on all the variables in a factor matrix, which effectively differentiates the original variables by extracted factor. Factor loadings show an increasing difference between lower weights, centering them closer to zero, and higher weights, converging them to one, therefore allowing an easier interpretation of the results. In this study, food items with factor loadings of ≥ |0.3| were considered influential and were used to define a descriptive ‘pattern’ of the diet associated with the factor. Factor scores for each subject were obtained by the regression method of Thomson 1951 [43]. Their associations with potential covariates were tested using Pearson correlation for numeric variables and *t*-test or ANOVA for categorical variables. Following checks to confirm that the metabolomics data met the corresponding assumptions, associations between the identified maternal dietary patterns and metabolite concentrations in cord blood were analyzed using linear regression, in four differently adjusted models. The first model was a “crude model”, minimally adjusted for potential batch effects in the metabolite measurements. The “main model” included further adjustment for city, sex, gestational age, smoking during the 3rd trimester, maternal education, and maternal age. Additional models were run, adjusting for covariates of the main model plus maternal pre-pregnancy BMI and gestational weight gain (“main model +”), and finally adjusting for all above-mentioned covariates as well as child’s birth weight (“main model ++”). The main model is considered the most important model for interpretation as maternal BMI, gestational weight gain, and child’s birth weight may lie in the causal pathway (being potentially affected by diet and in turn altering metabolic processes), and hence models with these covariates could be over-adjusted. Correction for multiple hypothesis testing via false discovery rate (FDR) was applied to all models to determine significant associations.

## 3. Results

### 3.1. Study Population

The present analysis comprises 739 participants from the Munich (77%) and Bad Honnef (23%) LISA study centers, for whom complete information on maternal diet and cord blood metabolites was available (representing 39% and 18% of newborns initially recruited from Munich and Bad Honnef, respectively). Basic characteristics of the study population are described in Table 1 with means (standard deviation) for numeric variables and frequencies (%) for categorical variables. The average age of the mothers was 32.0 ± 4.0 years, and ~75% of them had a normal BMI. Among the remaining 25%, 124 were overweight and 43 underweight, all together presenting a mean maternal BMI of 22.6 ± 4.1. On average, ~90% of the mothers reported to be non-smokers during the last trimester of pregnancy. Gestational weight gain averaged 0.3 ± 0.1 kg per month over approximately 40.0 ± 1.2 weeks. The offspring had a mean birth weight of 3.5 ± 0.4 kg.

### 3.2. Maternal Dietary Patterns

Following factor analysis, ten dietary patterns were retained, explaining 28% of the total variance of maternal diet. This level of total explained variance is expected for food-frequency questionnaires, including a large number of items, given the multidimensionality of diet, and is comparable in magnitude to that observed in other studies [44,45]. The dietary patterns were labeled based on the most relevant food items as indicated by the highest factor loadings. The chosen labels were hence data-dependent and entirely arbitrarily defined: Factor 1 = “vegetables”, Factor 2 = “fruits”, Factor 3 = “summer vegetables”, Factor 4 = “salad and dressings”, Factor 5 = “cereal, seeds, nuts, yoghurt, cheese”, Factor 6 = “butter vs. margarine”, Factor 7 = “meat and potato”, Factor 8 = “sweets”, Factor 9 = “fish and shellfish”, and Factor 10 = “seasonal”. For the ten dietary patterns (factors 1–10), the factor loadings for each food item and the percentage of variance explained are presented in Table 2. The foods with factor loadings of ≥|0.3|, which were used to label each dietary pattern, are marked with an asterisk, as well as separately listed in Supplementary Table S1. The dietary patterns indicated the tendency for mothers to have specific eating behaviors characterized by more (or less) of certain foods.

Some of the covariates presented significant associations with the resulting dietary patterns (Supplementary Table S2). In Bad Honnef, there was a higher maternal consumption of the “vegetables” and “meat and potato” patterns (*p* < 0.001) and, in contrast, lower intakes of “fruits”, “summer vegetables” and “butter vs. margarine” patterns (*p* < 0.05). Mothers who smoked during the last trimester of pregnancy also showed a difference in their diet. Non-smokers indicated a preference for the “cereal, nut, seeds, yoghurt, cheese” and the “butter vs. margarine” patterns (*p* < 0.001), whereas the smokers had higher intakes of the “meat and potato” dietary pattern (*p* < 0.05). High maternal education was associated with a higher consumption of “fruits” (*p* < 0.05), “cereal, nut, seeds, yoghurt, cheese” and “butter vs. margarine” (*p* < 0.001) patterns, with significantly less intakes of “meat and potato” (*p* < 0.001); the opposite was the case for the lowest educated mothers. An increase in “cereal, nut, seeds, yoghurt, cheese”, “butter vs. margarine” (*p* < 0.001), and “fish and shellfish” (*p* < 0.05) was also observed with increasing maternal age.

### 3.3. Associations of Dietary Patterns with Cord Blood Metabolites

Since the many linear regression associations tested cannot be displayed in a single table, only associations with *p* < 0.005 in at least one model are presented in Table 3. Associations that were significant following correction for multiple hypothesis testing are shown in bold and marked with an asterisk. The full list of all associations is provided in Supplementary Table S3. Generally, a positive beta coefficient indicates that a dietary pattern is proportionately influencing the level of a metabolite, and a negative beta would imply that metabolite levels are inversely associated with the dietary pattern.

A significant positive association was seen for the “cereal, nut, seeds, yoghurt, cheese” pattern with SM C30:1 in the crude model, which was also significant in model ++. For this dietary pattern, an association with SM C43:2 was also observed in the crude model, but this association was not statistically significant in any of the further-adjusted models. For the “butter vs. margarine” pattern, the crude model resulted in eight significant associations, including positive associations with SM C39:1, SM C43:2, LPCe C18:0, LPCe C16:0, and NEFA C15:0 and C17:0, and inverse associations with LPCa C18:3 and AC C8:1. After adjustment for covariates, none of these associations were statistically significant; however, effect sizes remained stable for AC C8:1 (*p* = 0.001), SM C39:1 (*p* = 0.001) and SM C43:2 (*p* = 0.003) after adjustment in the main model. A significant, direct association with AC C3:0 was observed for the “meat and potato” pattern across all models. “Fish and shellfish” presented an inverse association with n-6 osbond acid (C22:5 n6) across all models except model ++. Further, this pattern had a direct association with n-3 eicosapentaenoic acid (EPA, C20:5 n3) in the crude model, which approached significance in all further-adjusted models (with *p*-value = 0.001).

## 4. Discussion

In the present study, we investigated the association of maternal diet with a range of chemically characterized cord blood metabolites. Using factor analysis, we identified ten dietary patterns describing the eating behaviors of mothers during the last four weeks of pregnancy. Dietary patterns presenting robust associations with metabolites across differently adjusted models were “fish and shellfish” and “meat and potato”. For the dietary patterns “cereals, seeds, nuts, yoghurt, cheese” and “butter vs. margarine”, associations were observed which were stable across all models in terms of effect size, but which were not statistically significant beyond the crude model.

### 4.1. Fish and Shellfish

For “fish and shellfish”, an inverse association was observed with osbond acid, and a positive association was observed with EPA (although the latter was only significant in the crude model, the effect size was barely reduced in all further-adjusted models, and these all had *p* = 0.001). In particular, oily fish and seafood are rich sources of EPA and docosahexaenoic acid (DHA, C22:6 n3) [46], thus a positive association with EPA is to be expected. A significant positive association with DHA was however not observed in our analyses. Incorporation of DHA in membranes is reported to be slower and more erratic than that of EPA [47]. Some [48,49] but not all [50,51] studies have reported increased cord blood DHA levels with DHA supplementation during pregnancy. The importance of DHA for optimal neurodevelopment of the fetus is now widely recognized [52,53]; however, research on DHA during pregnancy was at very early stages during the time of dietary assessment (1995–1998) [48], and so residual confounding due to DHA supplementation is unlikely. It has been previously highlighted that only a small proportion of the variation in cord blood DHA levels is explained by maternal DHA intake [54]. It is postulated that in the last trimester of pregnancy there is a preferential transfer of DHA from the mother to the fetus, leading to a depletion of maternal stores [50]. Differences in dietary intake at this stage may hence have a more appreciable effect on maternal DHA status than on cord blood levels. In line with our results, a study of maternal diet in coastal and inland China reported higher EPA levels in cord blood in the coastal population, but no significant differences in DHA [55]. Finally, osbond acid is synthesized when there is a lack of DHA, and is hence considered a marker of DHA functional shortage [56]. The observed lower osbond acid related to “fish and shellfish” intake may thus provide a better indication of diet-induced changes to cord blood DHA status than DHA itself.

### 4.2. Meat and Potato

The dietary pattern “meat and potato” was significantly and consistently positively associated with AC C3:0 across all models. In preliminary descriptive analyses, this dietary pattern was correlated with maternal education and smoking during the third trimester (Table S2). However, adjusting for such variables in the main model did not reduce the magnitude of effect. The short-chain AC C3:0 is a by-product of branched-chain amino acid (BCAA) catabolism [57]. In line with our findings, observational studies in adults have shown this metabolite to be associated with higher meat intakes [58,59], and increased levels were observed in infants provided with high-protein formula milk [60]. Short-chain ACs have been associated with high birth weight [61], as well as with obesity and metabolic syndrome in adults [62]. Considering that meat is the richest dietary source of BCAA [63], these results add to the evidence suggesting an involvement of the BCAA degradation pathway in linking maternal diets high in red and processed meat to metabolic disturbances in the offspring [64,65]. We did not observe differences in levels of BCAA; however, the aromatic amino acid tryptophan was slightly increased in association with this dietary pattern (*p* = 0.002 in the main model). BCAA are known to elevate aromatic amino acid concentrations, suggested to be the most potent promoters of IGF-1 secretion [66], thereby enhancing infant weight gain according to the early protein hypothesis [67]. Finally, the observed inverse association with palmitoleate (C16:1n7), which was not significant but consistent across all models at *p* ≤ 0.002, may reflect reduced adipocyte de novo lipogenesis [68]. Palmitoleate from adipose tissue is suggested to promote insulin sensitivity [69,70], and lower levels could reflect unfavorable fetal metabolism. Indeed, the same opposing associations of AC C3:0 and palmitoleate have been previously reported in relation to type 2 diabetes mellitus and fasting glucose sensitivity [71]. It is suggested that chronic elevations in BCAA and related metabolites synergize with a rise in circulating fatty acids to drive a state of chronic hyperinsulinemia and metabolic disease [72]. Taken together, our results indicate that a maternal diet high in meat might already promote such metabolic disruptions in the offspring in utero.

### 4.3. Butter vs. Margarine

In relation to the “butter vs. margarine” pattern, an inverse association with AC C8:1 and positive associations with SM C39:1 and SM C43:2 were observed in the crude model, and although not statistically significant (*p* ≤ 0.003), the magnitude of these effects remained stable following further adjustment in the main model. The inverse association observed with AC C8:1 could be an indication of better mitochondrial function, as medium-chain ACs are suggested to result from a mismatch between beta-oxidation and tricarboxylic acid (TCA) cycle capacity [73] and have also been associated with gestational diabetes [74]. Indeed, this specific metabolite has been associated with increased body fat [75] and pre-diabetic conditions [76], so lower levels would suggest a beneficial effect in the offspring by consuming butter at the expense of margarine during pregnancy. A similar dietary pattern was described by Floegel et al. in adults, for which an inverse cross-sectional association with AC C8:1 was also observed [77].

The positive associations of butter with SM C39:1 and 43:2 have to our knowledge not been previously reported. Dairy products are important dietary sources of SM; however, during butter manufacture, the milk fat globule membrane that contains the SM is separated from the triglyceride-rich core and removed [78]. The associated metabolites are therefore more likely to be the result of the endogenous combination of ceramides and sphingomyelins with very long-chain saturated and monounsaturated fatty acids derived from fatty acid elongation [79,80]. Most saturated and monounsaturated very long-chain fatty acids are sphingolipid components and thus play important roles in skin barrier formation and neural functions [81]. Higher SM C39:1 has been observed within LDL in pregnant women [82], and cholesterol levels are known to increase during pregnancy [83]. LDL receptors in the placenta allow the uptake of LDL, providing important cholesterol and fatty acids for fetal development [84]. The direct associations observed in our study suggest a specific relevance of these metabolites for the fetus. Indeed, SM with saturated acyl chains are the main constituents of lipid rafts, and are important for membrane function and in the regulation of cell signaling pathways, especially in the brain [85]. The biological importance of SM for brain development has been shown in a trial that reported neurobehavioral benefits in preterm infants given formula enriched with SM [86].

### 4.4. Cereals, Nuts, Seeds, Yoghurt, Cheese

The association of “cereals, seeds, nuts, yoghurt, cheese” with SM C30:1 could be explained by the high intakes of dairy that characterize this dietary pattern. We expect that the fatty acyl chain in SM C30:1 is a medium-chain saturated fatty acid (MCFA, C12:0), assuming a sphingosine backbone (C18:1). Although most common in coconut milk, this fatty acid can also be found in bovine milk and is suggested to have antibacterial properties [87], as well as modulating cellular signaling and regulating key glucose and lipid metabolism [88]. Saturated MCFA are used in mitochondrial energy production independent of the carnitine transport system, thus allowing valuable ATP molecules to be saved for other cellular processes in the developing fetus [89]. Indeed, a study observed lower levels of MCFA in the diet of women giving birth to preterm and small for gestational age infants [90], highlighting their value for fetal development.

From the present findings, it seems that effects of maternal diet are overruled in some cases by the corresponding placental transfer mechanisms. In general, given the predominance of significant associations with phospholipids, our findings suggest that facilitated diffusion is of greater importance for these than for amino acids. Diffusion leads to proportional concentrations, whereas the active transport of amino acids works against the proportionality of maternal and cord blood concentrations (higher amino acid concentrations in fetal than in maternal circulation) [91], and may explain why maternal diet was reflected to a lesser extent in cord blood amino acids. In line with this principle is the lack of association for fish intake with DHA, which could be explained by the selective maternal–fetal transfer of DHA being more reliant on existing maternal stores than on dietary DHA intake [54]. Additionally, an unexpected observation is that associations between fish and long-chain PUFA were seen for GPL-FA but not for PC species. This could signify insufficient choline availability and thus limited phosphatidylethanolamine-N-methyltransferase activity, reducing placental transport of PCs carrying long-chain PUFA [92].

### 4.5. Strengths and Limitations

A major strength of this study is the cord blood metabolomics data available in a large sample of 739 participants, which entails a tremendous effort and is highly unusual. Our study provides a data-driven description of dietary patterns, defined on the basis of foods most commonly associated with one another (consumed together or at the expense of each other). This takes into account the complexities of habitual diet, such as potential interactions or synergistic effects within the food complex or correlations among different foods and nutrients, the individual effects of which are often difficult to disentangle. This method also ensures enough power in the analysis of associations with a large number of metabolites, whereas a focus on individual foods would signify a much larger number of tests and an increased potential for chance findings. Total explained variance by the different factors was indeed low; however, this is not uncommon for factor analysis of dietary data given its multidimensionality, and is comparable to that observed in other studies [44].

The applied method for dietary assessment entails some limitations. A non-quantitative FFQ was used, which was part of the main questionnaire completed upon recruitment, and was not previously validated. The lack of more detailed portion size information limits our ability to precisely estimate associations of metabolites with intake levels of individual foods. Thus, with the available data, we cannot show which specific food items and intake levels within the derived dietary patterns underlie the observed associations. The FFQ was however not intended for an estimation of exact quantities, or nutrient content of the reported diet. Rather, the aim was to provide a general estimation of dietary intakes of common foods to allow the ranking of individual intakes with respect to the overall study population, for which frequency alone should provide sufficient information [93]. Potential reporting errors common to all dietary assessment methods may however limit the accuracy of the collected dietary data. On the other hand, we cannot know to what extent reports of diet during the last four weeks of pregnancy are representative of usual diet. Despite this, we observed plausible and robust associations between dietary patterns and a select few metabolites. Nonetheless, residual confounding cannot be ruled out and given the cross-sectional nature of the study, a causal effect of diet cannot be assumed. We also cannot exclude possible variation arising due to the length of time until storage of samples or the duration of storage [29].

## 5. Conclusions

This study demonstrated clear associations between maternal dietary patterns consumed during pregnancy and cord blood metabolites. Significant associations were observed specifically in relation to a pattern high in fish and shellfish and to a pattern high in meat and potatoes. The identified associated metabolites highlight possible mechanisms through which consuming certain types of dietary patterns during pregnancy may influence both short- and long-term health of the offspring. In this context, the relevance of lipid signaling, beta-oxidation, and branched-chain amino acid degradation are discussed. This study provides novel findings that, if confirmed in further studies, could greatly aid our understanding of the role of diet during pregnancy and hence improve dietary recommendations. Our findings also indicate the great potential for establishing biomarkers for dietary patterns of pregnant women, which could overcome the inherent imprecision of current available methods of assessing dietary intakes.

## Figures and Tables

**Table 1:** Study population characteristics.

	N	n (%) or Mean ± SD
Maternal age (years)	739	32.4 ± 4.1
Maternal, pre-pregnancy BMI (kg/m^2^)	725	22.5 ± 4.1
Overweight (≥25)	725	124 (17.1)
Normal (≥18.5 and <25)	725	558 (77)
Underweight (<18.5)	725	43 (5.9)
Maternal education		
Low (<10 years)	733	71 (9.7)
Medium (10 years)	733	232 (31.7)
High (>10 years)	733	430 (58.7)
Gestational age (weeks)	730	40 ± 1.2
Gestational weight gain (kg/month)	715	0.4 ± 0.1
Birth weight (kg)	739	3.5 ± 0.4
City		
Munich	739	568 (76.9)
Bad Honnef	739	171 (23.1)
Sex		
Female	739	395 (53.5)
Male	739	344 (46.5)
Smoking, during 3rd trimester		
Yes	705	74 (10.5)
No	705	631 (89.5)

**Table 2:** Food item factor loadings from factor analysis.

Food Items in FFQ	Factor 1	Factor 2	Factor 3	Factor 4	Factor 5	Factor 6	Factor 7	Factor 8	Factor 9	Factor 10
Milk, buttermilk					0.183					
Yoghurt		0.203			0.388 *					
Cheese		0.129			0.406 *		0.146			
Cream, sour cream, crème fraiche, coffee cream				0.226	0.124	0.172	0.214	0.138		
Butter						0.834 *				
Margarine				−0.155		−0.679 *	0.241			
Vegetable oil (not olive)				0.349 *	0.179		0.109			
Oily fruits and seeds				0.194	0.400 *		−0.140		0.212	
Vegetable cooking fat							0.146			
Nuts	0.116				0.334 *				0.131	0.238
Chocolate							0.206	0.287	−0.148	0.218
Liver									0.179	
Liver sausage, pate				−0.101			0.368 *			
Pork							0.488 *	0.109		
Fish	0.152			0.133	0.203		0.225		0.398 *	
Seafood, shellfish				0.124			−0.109		0.490 *	
Canned fish, smoked fish		0.122					0.192		0.332 *	
Boiled potatoes	0.391 *				0.109		0.393 *			
Fried potatoes, chips	0.116			0.147	−0.129		0.226	0.245		
Vegetable juice	0.234	0.135		−0.115					0.198	
Raw carrots	0.358 *	0.172	0.154		0.254		−0.101	−0.131		
Carrots	0.659 *	0.136								
Spinach, Swiss chard	0.471 *								0.183	
Cooked vegetables	0.482 *						0.198			
Celery	0.304 *			0.132		0.112			0.212	0.113
Vegetables	0.370 *		0.117					0.169		
Raw tomatoes		0.156	0.479 *	0.186	0.219				0.119	−0.222
Raw sweet pepper		0.140	0.887 *	0.107	0.137					
Cooked sweet pepper	0.285		0.500 *					0.109	0.141	
Lettuce		0.112	0.158	0.719 *	0.141	0.145	−0.109			
Mayonnaise, salad dressing				0.637 *						
Juices		0.363 *								
Citrus fruits		0.547 *							0.132	0.339 *
Apple	0.158	0.441 *			0.243					
Kiwi, pineapple, mango	0.182	0.547 *			0.115				0.168	
Banana	0.108	0.463 *			0.200					−0.103
Strawberry		0.168	0.159					0.234	0.106	−0.493 *
Fruit syrup, juice concentrate								0.233		
Cake								0.357 *		0.150
Fruit cake							0.116	0.478 *		
Gingerbread (Lebkuchen)		0.131							0.101	0.486 *
Sweet dairy foods					0.143			0.460 *		−0.144
Eggs					0.152		0.272	0.114		
Soy milk, soy products					0.108		−0.110		0.105	
Cereals		0.145		0.131	0.468 *		−0.202			
Variance Explained (%)	3.9	3.3	3.2	3.1	3.0	2.9	2.6	2.2	2.0	1.9
Cumulative Variance (%)	3.9	7.2	10.4	13.5	16.5	19.4	22.0	24.2	26.2	28.2

Values less than 0.1 were omitted in order to observe noteworthy factor loadings. * Factor loadings ≥ |0.3|. Factor 1 = Vegetables; Factor 2 = Fruits; Factor 3 = Summer vegetables; Factor 4 = Salad and dressings; Factor 5 = Cereal, seeds, nuts, yoghurt, cheese; Factor 6 = Butter vs. margarine; Factor 7 = Meat and potato; Factor 8 = Sweets; Factor 9 = Fish and shellfish; Factor 10 = Seasonal.

**Table 3:** Association of dietary patterns with cord blood metabolites.

		Crude Model				Main Model				Main Model +				Main Model ++			
Dietary Pattern	Metabolite	n	beta	se	*p*	n	beta	se	*p*	n	beta	se	*p*	n	beta	se	*p*
**Vegetables**	PCa C38:5	734	0.056	0.017	0.001	689	0.060	0.017	0.001	674	0.053	0.017	0.003	674	0.052	0.018	0.003
	PCe C36:3	735	0.068	0.020	0.001	690	0.071	0.021	0.001	675	0.067	0.021	0.002	675	0.065	0.021	0.002
	PCe C36:4	737	0.060	0.018	0.001	692	0.063	0.019	0.001	677	0.057	0.019	0.003	677	0.056	0.019	0.004
	Val	734	0.033	0.011	0.004	689	0.038	0.012	0.001	674	0.039	0.012	0.001	674	0.039	0.012	0.001
	His	737	0.055	0.015	<0.001	692	0.051	0.016	0.001	677	0.044	0.016	0.006	677	0.041	0.016	0.009
	C22:5 n6	734	0.066	0.021	0.002	690	0.066	0.022	0.002	675	0.061	0.022	0.006	675	0.056	0.022	0.010
	Lys	610	0.040	0.015	0.007	573	0.042	0.015	0.005	560	0.037	0.015	0.013	560	0.038	0.015	0.013
	PCe C34:1	737	0.059	0.019	0.002	692	0.056	0.020	0.006	677	0.050	0.021	0.015	677	0.049	0.021	0.018
	SMa C30:1	586	0.106	0.034	0.002	550	0.093	0.035	0.008	539	0.086	0.036	0.017	539	0.088	0.036	0.014
**Fruits**	PCa C42:1	385	−0.121	0.033	<0.001	365	−0.109	0.034	0.002	357	−0.104	0.035	0.003	357	−0.105	0.035	0.003
	SMa C42:5	434	−0.112	0.039	0.004	410	−0.122	0.041	0.003	404	−0.116	0.041	0.005	404	−0.117	0.041	0.005
	H1	719	−0.169	0.065	0.010	674	−0.186	0.068	0.006	659	−0.209	0.069	0.002	659	−0.206	0.069	0.003
	C20:4 n6	732	−0.036	0.012	0.003	688	−0.034	0.013	0.007	673	−0.035	0.013	0.006	673	−0.035	0.013	0.006
	AC C10:1	650	−0.084	0.027	0.002	616	−0.075	0.028	0.008	602	−0.073	0.029	0.011	602	−0.075	0.029	0.009
	C18:2 n6	733	−0.040	0.014	0.006	689	−0.037	0.015	0.014	674	−0.042	0.015	0.007	674	−0.044	0.015	0.004
	LPCa C20:4	735	−0.071	0.021	0.001	690	−0.052	0.022	0.017	675	−0.052	0.022	0.018	675	−0.046	0.022	0.032
	Met	736	0.047	0.017	0.004	691	0.040	0.017	0.019	676	0.037	0.017	0.032	676	0.036	0.017	0.035
	PCa C38:4	737	−0.052	0.018	0.004	692	−0.042	0.018	0.024	677	−0.040	0.019	0.032	677	−0.042	0.019	0.026
**Summer**	PCa C40:5	738	−0.065	0.023	0.004	693	−0.067	0.023	0.004	678	−0.064	0.024	0.007	678	−0.062	0.023	0.008
**Vegetables**	PCe C38:3	730	−0.076	0.024	0.001	685	−0.067	0.024	0.006	670	−0.063	0.024	0.011	670	−0.064	0.024	0.009
	PCe C38:2	726	−0.093	0.032	0.004	681	−0.081	0.032	0.012	666	−0.076	0.032	0.020	666	−0.076	0.032	0.019
**Salad**	Gln	715	−0.097	0.027	<0.001	674	−0.081	0.028	0.004	660	−0.077	0.028	0.006	660	−0.077	0.028	0.006
**and**	LPCa C18:2	736	0.068	0.022	0.002	691	0.061	0.022	0.006	676	0.064	0.023	0.005	676	0.068	0.022	0.002
**Dressings**	C18:2 n6	733	0.047	0.014	0.001	689	0.040	0.015	0.007	674	0.038	0.015	0.014	674	0.037	0.015	0.016
	AC C16:0	738	−0.085	0.028	0.003	693	−0.077	0.029	0.008	678	−0.067	0.030	0.024	678	−0.067	0.030	0.024
	PCa C36:2	735	0.064	0.019	0.001	690	0.052	0.020	0.010	675	0.053	0.021	0.010	675	0.053	0.021	0.011
	LPCa C18:3	604	0.092	0.035	0.009	576	0.087	0.036	0.016	564	0.095	0.036	0.010	564	0.105	0.036	0.004
	Orn	736	−0.064	0.022	0.004	691	−0.040	0.021	0.056	676	−0.043	0.021	0.045	676	−0.043	0.021	0.044
	Gly	738	−0.048	0.017	0.005	693	−0.032	0.017	0.059	678	−0.031	0.017	0.070	678	−0.031	0.017	0.070
	Met	736	−0.051	0.017	0.002	691	−0.030	0.017	0.071	676	−0.030	0.017	0.078	676	−0.030	0.017	0.075
**Cereals,**	SMa C30:1	586	0.128	0.036	<0.001 *	550	0.136	0.038	<0.001	539	0.141	0.039	<0.001	539	0.143	0.039	<0.001 *
**Seeds, Nuts,**	SMa C43:2	689	0.087	0.025	<0.001 *	650	0.080	0.027	0.003	637	0.074	0.027	0.007	637	0.074	0.027	0.007
**Yogurt,**	AC C2:0	735	−0.073	0.022	0.001	690	−0.065	0.024	0.006	675	−0.061	0.024	0.012	675	−0.061	0.024	0.012
**Cheese**	SMa C35:1	737	0.056	0.019	0.004	692	0.057	0.021	0.008	677	0.051	0.021	0.017	677	0.051	0.021	0.017
	SMa C33:1	737	0.055	0.019	0.004	692	0.051	0.020	0.013	677	0.046	0.021	0.026	677	0.046	0.021	0.026
	LPCe C16:0	692	0.080	0.026	0.002	654	0.060	0.028	0.032	641	0.054	0.028	0.056	641	0.052	0.028	0.061
**Butter**	AC C8:1	736	−0.143	0.038	<0.001 *	692	−0.142	0.041	0.001	677	−0.120	0.042	0.004	677	−0.118	0.042	0.005
**vs.**	SMa C39:1	734	0.078	0.023	0.001 *	689	0.081	0.024	0.001	674	0.075	0.025	0.003	674	0.076	0.025	0.002
**Margarine**	NEFA C22:3	565	−0.054	0.026	0.035	534	−0.081	0.027	0.002	523	−0.070	0.027	0.010	523	−0.071	0.027	0.010
	SMa C43:2	689	0.079	0.025	0.002 *	650	0.080	0.027	0.003	637	0.074	0.027	0.007	637	0.074	0.027	0.007
	LPCe C18:0	733	0.098	0.026	<0.001 *	688	0.080	0.028	0.004	673	0.066	0.028	0.019	673	0.066	0.028	0.019
	C15:1	725	0.077	0.027	0.004	681	0.080	0.028	0.004	666	0.066	0.028	0.020	666	0.067	0.028	0.019
	LPCa C18:3	604	−0.117	0.036	0.001 *	576	−0.106	0.038	0.005	564	−0.118	0.039	0.002	564	−0.122	0.038	0.001
	LPCe C16:0	692	0.092	0.026	0.001 *	654	0.072	0.028	0.010	641	0.072	0.028	0.011	641	0.071	0.028	0.012
	NEFA C17:0	734	0.076	0.021	<0.001 *	690	0.057	0.022	0.011	675	0.057	0.023	0.012	675	0.058	0.023	0.010
	NEFA C15:0	734	0.110	0.032	0.001 *	690	0.080	0.033	0.016	675	0.077	0.034	0.023	675	0.078	0.034	0.022
	NEFA C19:1	736	0.079	0.026	0.002	691	0.060	0.027	0.028	676	0.061	0.028	0.027	676	0.062	0.028	0.025
**Meat**	AC C3:0	737	0.110	0.026	<0.001 *	692	0.127	0.027	<0.001 *	677	0.122	0.027	<0.001 *	677	0.122	0.027	<0.001 *
**and**	C16:1 n7	732	−0.052	0.016	0.001	689	−0.057	0.017	0.001	674	−0.054	0.018	0.002	674	−0.054	0.018	0.002
**Potato**	Trp	733	0.042	0.014	0.002	688	0.045	0.015	0.002	673	0.043	0.015	0.004	673	0.043	0.015	0.004
	Gln	715	−0.089	0.029	0.003	674	−0.060	0.030	0.045	660	−0.057	0.030	0.060	660	−0.057	0.030	0.060
	C18:1 n9	732	−0.033	0.011	0.003	688	−0.033	0.012	0.007	673	−0.034	0.012	0.006	673	−0.034	0.012	0.006
	SMa C32:2	737	−0.073	0.025	0.004	692	−0.054	0.026	0.039	677	−0.045	0.027	0.087	677	−0.044	0.026	0.093
**Sweets**	NEFA C24:2	540	−0.066	0.022	0.003	505	−0.069	0.024	0.004	494	−0.065	0.024	0.007	494	−0.068	0.024	0.004
**Fish**	C22:5 n6	734	−0.084	0.022	<0.001 *	690	−0.099	0.024	<0.001 *	675	−0.095	0.024	<0.001 *	675	−0.086	0.024	<0.001
**and**	C20:5 n3	729	0.118	0.028	<0.001 *	685	0.105	0.031	0.001	670	0.109	0.032	0.001	670	0.103	0.031	0.001
**Shellfish**	C22:6 n3	734	0.051	0.017	0.003	690	0.048	0.018	0.008	675	0.050	0.018	0.007	675	0.047	0.018	0.011
	AC C18:1	693	0.091	0.032	0.004	655	0.083	0.033	0.013	642	0.077	0.033	0.021	642	0.075	0.033	0.025
	NEFA C12:0	736	−0.119	0.042	0.005	691	−0.098	0.046	0.034	676	−0.091	0.046	0.049	676	−0.096	0.046	0.039
**Seasonal**	NEFA C22:2	635	0.074	0.024	0.003	598	0.086	0.025	0.001	586	0.091	0.025	<0.001	586	0.091	0.025	<0.001
	Ser	736	0.057	0.020	0.004	691	0.068	0.020	0.001	676	0.069	0.020	0.001	676	0.069	0.020	0.001
	NEFA C26:3	637	0.053	0.021	0.011	598	0.064	0.021	0.003	585	0.061	0.021	0.004	585	0.061	0.021	0.004
	NEFA C20:2	643	0.071	0.029	0.015	606	0.086	0.030	0.004	593	0.091	0.030	0.003	593	0.091	0.030	0.003
	C20:1 n9	694	0.079	0.027	0.003	651	0.073	0.028	0.008	636	0.070	0.028	0.013	636	0.070	0.028	0.013

Values are presented as beta coefficients (beta) and their corresponding standard error (se). Crude model: adjusted for batch; Main model: Crude model further adjusted for city, sex, gestational age, smoking, maternal education, and maternal age; Main model +: Main model further adjusted for maternal BMI and maternal gestational weight gain; Main model ++: Main model + further adjusted for birth weight. *p* = *p*-value. * Significant *p*-values after FDR correction for multiple hypothesis testing are highlighted in bold and marked with an asterisk.

## Data Availability

Restrictions apply to the availability of these data. The datasets generated and/or analyzed during the current study are not publicly available due data protection but are available from the corresponding author on reasonable request and acceptance of a data transfer agreement from the legal department of the Helmholtz Zentrum München.

## Supplementary Materials

The following supporting information can be downloaded at: https://www.mdpi.com/article/10.3390/biom12101333/s1, Table S1: List of foods with factor loadings of ≥|0.3| used to label dietary patterns; Table S2: Association between dietary patterns and covariates; Table S3: Association of dietary patterns with cord blood metabolites (all results).
