# Supplementary material for: Association of Maternal Diet during Pregnancy and Metabolite Profile in Cord Blood
Source: Biomolecules. 2022 Sep 21;12(10):1333. doi: 10.3390/biom12101333 (PMC9599655; doi:10.3390/biom12101333)
Supplement: Supplementary file 1 [file biomolecules-12-01333-s001.zip › Supplementary_Table_S1.pdf]

Table S1. List of foods with factor loadings of ≥ |0.3| used to label dietary patterns

Factor	Dietary Pattern Label	Foods	
		Higher intakes	Lower intakes
1	Vegetables	Boiled potatoes<br>Raw carrots<br>Carrots<br>Spinach, Swiss chard<br>Cooked vegetables<br>Celery<br>Vegetables	
2	Fruits	Juices<br>Citrus fruits<br>Apple<br>Kiwi, pineapple, mango<br>Banana	
3	Summer vegetables	Raw tomatoes<br>Raw sweet pepper<br>Cooked sweet pepper	
4	Salad & dressings	Vegetable oil (not olive)<br>Lettuce<br>Mayonnaise, salad dressing	
5	Cereal, seeds, nuts, yoghurt, cheese	Yoghurt<br>Cheese<br>Oily fruits and seeds<br>Nuts<br>Cereals	
6	Butter vs. margarine	Butter	Margarine
7	Meat & potato	Liver sausage, pate<br>Pork<br>Boiled potatoes	
8	Sweets	Cake<br>Fruit cake<br>Sweet dairy foods	
9	Fish & shellfish	Fish<br>Seafood, shellfish<br>Canned fish, smoked fish	
10	Seasonal	Citrus fruits<br>Gingerbread (Lebkuchen)	Strawberry
