# Supplementary material for: Association of Maternal Diet during Pregnancy and Metabolite Profile in Cord Blood
Source: Biomolecules. 2022 Sep 21;12(10):1333. doi: 10.3390/biom12101333 (PMC9599655; doi:10.3390/biom12101333)
Supplement: Supplementary file 1 [file biomolecules-12-01333-s001.zip › Supplementary_Table_S2.pdf]

Table S2: Association between Dietary patterns and Covariates

Covariates	Vegetables	Fruits	Summer Vegetables	Salad & Dressings	Cereal, Seeds, Nuts, Yogurt, Cheese	Butter vs Magarine	Meat & Potato	Sweets	Fish & Shellfish	Seasonal
City										
Munich, mean	**-0.14****	**-0.01***	**0.03***	0.29	0.09	**0.33****	**-0.22****	-0.04	0.03	**0.09***
Bad Honnef, mean	**0.12****	**-0.20***	**-0.15***	0.42	-0.03	**-0.10****	**0.28****	-0.07	-0.09	**-0.11***
Sex										
male, mean	-0.08	-0.05	0.01	0.34	0.08	0.25	-0.09	-0.04	0.01	0.01
female, mean	-0.07	-0.06	-0.03	0.31	0.04	0.21	-0.11	-0.05	-0.01	0.09
Smoking, during 3rd trimester										
no, mean	-0.07	**-0.02***	-0.01	0.31	**0.13****	**0.27****	**-0.12***	-0.05	<0.01	0.05
yes, mean	-0.20	**-0.28***	-0.03	0.49	**-0.32****	**-0.09****	**0.06***	-0.02	<0.01	0.03
Maternal education										
low, mean	-0.04	**-0.21***	-0.07	0.40	**-0.38****	**-0.10****	**<0.01****	-0.11	-0.01	-0.10
medium, mean	-0.06	**-0.12***	-0.03	0.34	**-0.09****	**0.11****	**0.04****	0.03	-0.03	0.01
high, mean	-0.09	**0.01***	0.01	0.30	**0.22****	**0.35****	**-0.20****	-0.08	0.02	0.10
Maternal age, correlation	0.05	0.04	0.06	-0.03	**0.14****	**0.16****	-0.02	-0.06	**0.12***	-0.01
Maternal pre-pregnancy BMI, correlation	**-0.08***	**-0.10***	<0.01	-0.05	**-0.13****	**-0.16****	**0.14****	0.04	0.04	0.01
Maternal gestational weight gain, correlation	-0.01	0.02	<0.01	0.03	-0.05	-0.05	**-0.12***	0.02	<0.01	<0.01
Birth weight, correlation	0.03	-0.03	-0.05	-0.04	0.03	<0.01	-0.02	-0.02	-0.04	-0.01
Gestational age, correlation	-0.04	0.01	-0.07	-0.01	0.03	-0.05	<0.01	-0.02	-0.01	0.03

* p<0.05 **p<0.001, p values are obtained from Pearson correlation for numeric variables, t-Test / ANOVA for categorical variables
